# Boron-Implanted Black Silicon Photodiode with Close-to-Ideal Responsivity from 200 to 1000 nm

**DOI:** 10.1021/acsphotonics.2c01984

**Published:** 2023-05-22

**Authors:** Olli E. Setälä, Kexun Chen, Toni P. Pasanen, Xiaolong Liu, Behrad Radfar, Ville Vähänissi, Hele Savin

**Affiliations:** † Department of Electronics and Nanoengineering, Aalto University, Tietotie 3, FI-02150 Espoo, Finland

**Keywords:** photodiode, UV detection, black silicon, ion implantation, responsivity

## Abstract

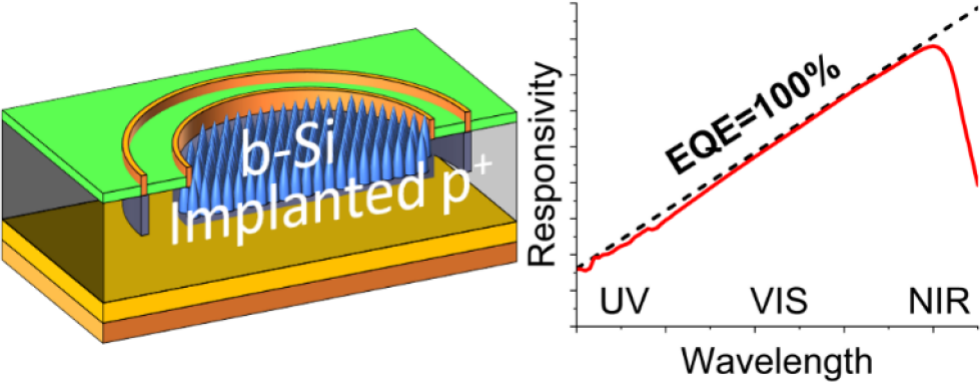

Detection of UV light has traditionally been a major challenge for Si photodiodes due to reflectance losses and junction recombination. Here we overcome these problems by combining a nanostructured surface with an optimized implanted junction and compare the obtained performance to state-of-the-art commercial counterparts. We achieve a significant improvement in responsivity, reaching near ideal values at wavelengths all the way from 200 to 1000 nm. Dark current, detectivity, and rise time are in turn shown to be on a similar level. The presented detector design allows a highly sensitive operation over a wide wavelength range without making major compromises regarding the simplicity of the fabrication or other figures of merit relevant to photodiodes.

## Introduction

Doped pn junction Si photodiodes occupy a major share of the photodiode market due to several of their unrivaled properties. Their low noise, high speed, easy and well-known processing techniques, compact sizes, and low cost often make them the preferred option for light detection in various applications such as optical communication systems,^1^ astronomical experiments,^2^ and medical imaging instruments.^3^ However, one area where doped pn junction photodiodes still struggle is the detection of UV light. Responsivity of the detectors has so far been limited by reflectance, whose impact is pronounced with shorter wavelengths.^4,5^ In fact, the commercial doped junction UV photodiodes still yield similar responsivity (e.g., ∼0.13 A/W at 250 nm, which corresponds to ∼65% quantum efficiency^4^) as they did already decades ago.

In solar cells, the reflectance losses have been eliminated by using a silicon surface with micro/nanostructures or so-called black silicon (b-Si),^6^ which in combination with effective surface passivation is already in industrial use. However, due to an increased surface area, external doping of b-Si leads to a high concentration of dopants inside the nanostructures, which in turn causes high Auger recombination.^7−9^ This is especially highlighted in the UV wavelengths, since UV photons are absorbed very close to the Si surface (within ∼100 nm) and need to diffuse to the depletion region without recombining in order to be collected.^4,5^ Recently, the increased recombination in nanostructured solar cells has been solved by developing recombination-free implanted^10^ and diffused^11^ pn junctions. As a result of these inventions, the external quantum efficiency (EQE) of the doped junction Si solar cells has been pushed to record values over a wide wavelength range. Since photodiodes are operationally very close to solar cells, this raises an interesting question if these methods could be applied to photodiodes, too.

In this Article, our aim is to employ some of the above approaches, i.e., nanostructures with an optimized implanted pn junction, in PIN photodiodes and study the resulting performance. We compare the photoresponsivity of our detector with that of the market leader diffused junction Si detectors, especially focusing on near and middle UV wavelengths of 200–400 nm. Furthermore, we systematically report and discuss the dark current, detectivity, and rise time.

## Methods

**Figure 1:**
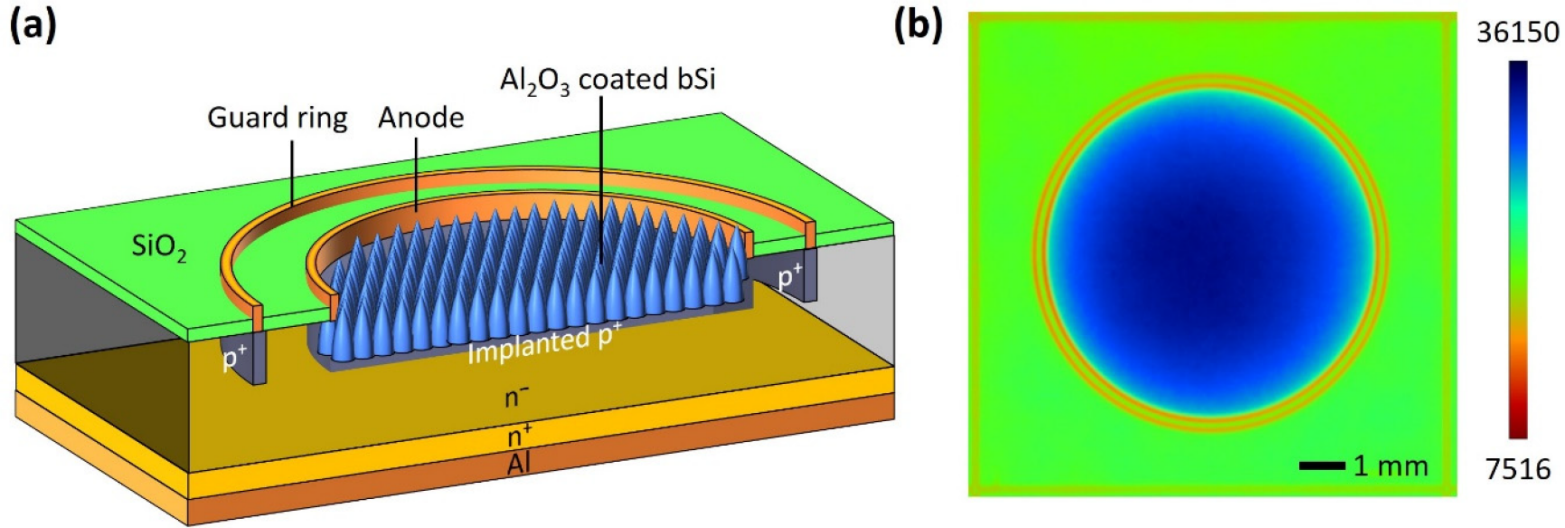
(a) Cross-section of the B-implanted b-Si photodiode. (b) Measurement of the recombination activity of a single detector chip using PL imaging. The units are arbitrary but demonstrate good uniformity on the active and surrounding areas.

In this work, the starting wafers were 4-in. lightly doped n-type FloatZone Si with a resistivity of >10 kOhm cm, a 350 μm thickness, and (111) orientation. A high-quality substrate with a minimal number of intrinsic impurities was chosen to allow the fabrication of a PIN photodiode with low bulk recombination and dark current. Figure 1a presents the cross-section of the final device. The circular active area of the chips is limited inside the anode ring and has a radius of 2.5 mm. The detection area is insulated from the surroundings by a circular guard ring, which prevents the outside leakage current from reaching the anode and thus helps to reduce the dark current.

Fabrication started by growing a thick (650 nm) silicon oxide on the front side of the wafer at 1000 °C in an H_2_O atmosphere to serve as a mask material during the following processes, but also as a passivation film outside of the active area on the final device (Figure 1a). Next, the oxide was patterned with photolithography to form the openings for the active areas. Cryogenic inductively coupled plasma–reactive ion etching (ICP–RIE) was employed to form the b-Si to the openings using a process described elsewhere.^12^ After b-Si formation, the oxide was again selectively etched to form the openings for guard rings. Next, the low recombination front junction was formed by applying boron implantation (10 keV implantation energy and 3 × 10^15^ cm^–2^ dose) and annealing parameters previously used in high-efficiency b-Si solar cells.^10,13^ Boron was also implanted outside the active area and phosphorus on the rear side of the photodiode to enable the formation of Ohmic contacts for the guard ring and the rear contact, respectively (see Figure 1a). Implantation was followed by a 20 min drive-in anneal at 1050 °C in an O_2_ atmosphere, after which the resulting thin oxide was removed from the implanted areas.

In ion implantation, the amount and depth of dopants is not dependent on the surface area, which makes it possible to create junctions with a low dopant concentration within the b-Si nanostructures. Conversely, employing a traditionally used diffusion doping technique would result in a high concentration of dopants inside the nanostructures and consequently vastly increase Auger recombination. The applied combination of front junction boron implantation and annealing parameters has indeed been shown to result in a low recombination junction with b-Si,^13^ which is the key for efficient UV light detection. A junction depth of ∼1.5 μm has been measured from a planar sample with a similar implantation to our device,^13^ and we assume it to roughly apply to our b-Si junction as well. Sheet resistance of the active area measured from our device by four-point probe was 85 Ω/sq. The nanostructured surface was then passivated by a 50 nm atomic layer deposited (ALD) aluminum oxide (Al_2_O_3_) film grown at 200 °C with trimethylaluminum and water as the precursors.

The metal contacts were formed by sputtering 300 and 1000 nm Al layers on the front and the rear side, respectively. Finally, the wafer was annealed in forming gas at 425 °C for 30 min. This final annealing served three purposes: (i) activation of Al_2_O_3_ passivation, (ii) sintering of Al contacts, and (iii) annealing of SiO_2_ with an Al layer on top (also known as Al-nealing). Al-nealing is known to greatly enhance the passivation performance of SiO_2_ on Si by creating atomic hydrogen to neutralize interface defects.^14,15^ Before the final annealing, Al film was removed from the top of the active area since Al-nealing has instead been shown to decrease the passivation performance of Al_2_O_3_.^16^ At the end, aluminum was etched off from the top of SiO_2_ to achieve the structure shown in Figure 1a.

The most important photodiode parameters were characterized from the finished devices. EQE was measured between 200 and 1100 nm with a 10 nm interval at zero bias, with measurement details described in ref (17). Spectral responsivity (*R*_λ_) was calculated from the EQE with
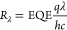
where *q* is the elementary charge, λ is the wavelength of light, *h* is the Planck constant, and *c* is the speed of light. *I*–*V* and *C*–*V* characteristics of the photodiodes were determined with a Hewlett-Packard Model 4145A Semiconductor Parameter Analyzer and a Hewlett-Packard Model 4192A LF Impedance Analyzer, respectively. A 100 kHz measurement frequency was used in the *C*–*V* measurement. The speed of the photodiodes was evaluated by a rise time measurement described in ref (18) and conducted under 405, 655, and 980 nm laser excitation and 5 and 10 V reverse biases. Photoluminescence (PL) imaging with a BT Imaging LIS-R2+ was utilized to demonstrate the uniformity of the recombination activity within the active area as well as outside it. Higher values represent lower recombination, but areas with different reflectances, i.e., active areas and surroundings, cannot be directly compared with each other.^19^ Matching values across all SiO_2_ areas show that good passivation can be achieved between the anode and the guard ring as that obtained in the areas outside the contact rings (Figure 1b). This is especially important to mitigate the dark current. Finally, dark current shot noise limited specific detectivity (D*) was calculated with^20,21^
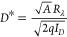
where *A* is the surface area of the detector and *I*_D_ is the dark current under specified reverse bias. We expect the dark current to be the dominating noise source when the photodiode is reverse biased. However, the calculation only gives the fundamental maximum achievable value of D*, since other noise sources, such as Johnson noise and 1/*f* (flicker) noise are not considered.

## Results and Discussion

**Figure 2:**
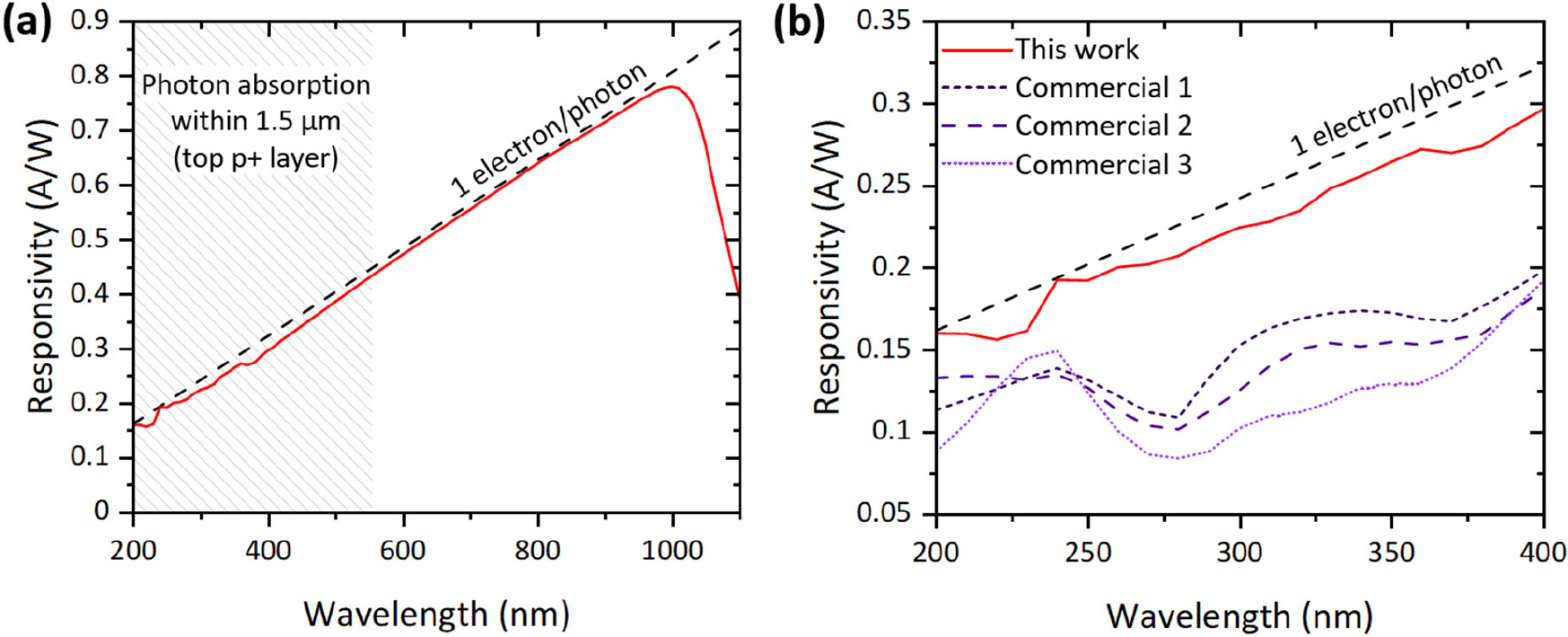
(a) Responsivity of a boron-implanted b-Si detector over a wide wavelength range measured at 0 V bias. Dashed line represents one collected electron per a photon. 1/e photon absorption limit for 1.5 μm junction is highlighted. (b) UV responsivity of market leader diffused Si UV photodiodes^23−25^ compared to this work.

Figure 2a shows the responsivity of our photodiode between 200 and 1100 nm. It can immediately be seen that the performance is near ideal over almost the whole wavelength range. The fact that the responsivity closely follows the line depicting the collection of one electron per each incoming photon implies that the photodiode is nearly free of reflectance and recombination losses. Such an excellent performance is achieved by mitigating the reflectance with b-Si and efficiently passivating the nanostructured surface with ALD Al_2_O_3_. Interestingly, responsivity remains high at wavelengths <550 nm, which correspond to absorption depths of <1.5 μm on the Si surface. These depths are related to our estimated p+ implantation depth of 1.5 μm, and thus, the high responsivity with these wavelengths proves that junction recombination is also minimal and the device is able to collect charge carriers generated at the very surface of it. This is also assisted by the negative fixed charge in the Al_2_O_3_ film repelling minority charge carriers away from the surface/implanted area.^22^

Figure 2b focuses specifically on UV responsivity (200–400 nm) and presents a comparison between our device and commercial diffused Si UV photodiodes,^23−25^ hereafter referred to as “Commercial UV photodiode 1/2/3”. Reference devices with the highest UV responsivity to the best of our knowledge were chosen. Interestingly, the commercial devices exhibit rather poor performance and clearly fall below our photodiode in that regard. Furthermore, the responsivity of the commercial devices remains far from ideal outside UV, too (not shown here). The combination of b-Si and optimized implanted junction instead allows near-ideal responsivity in UV, but also all the way up to near-infrared.

**Figure 3:**
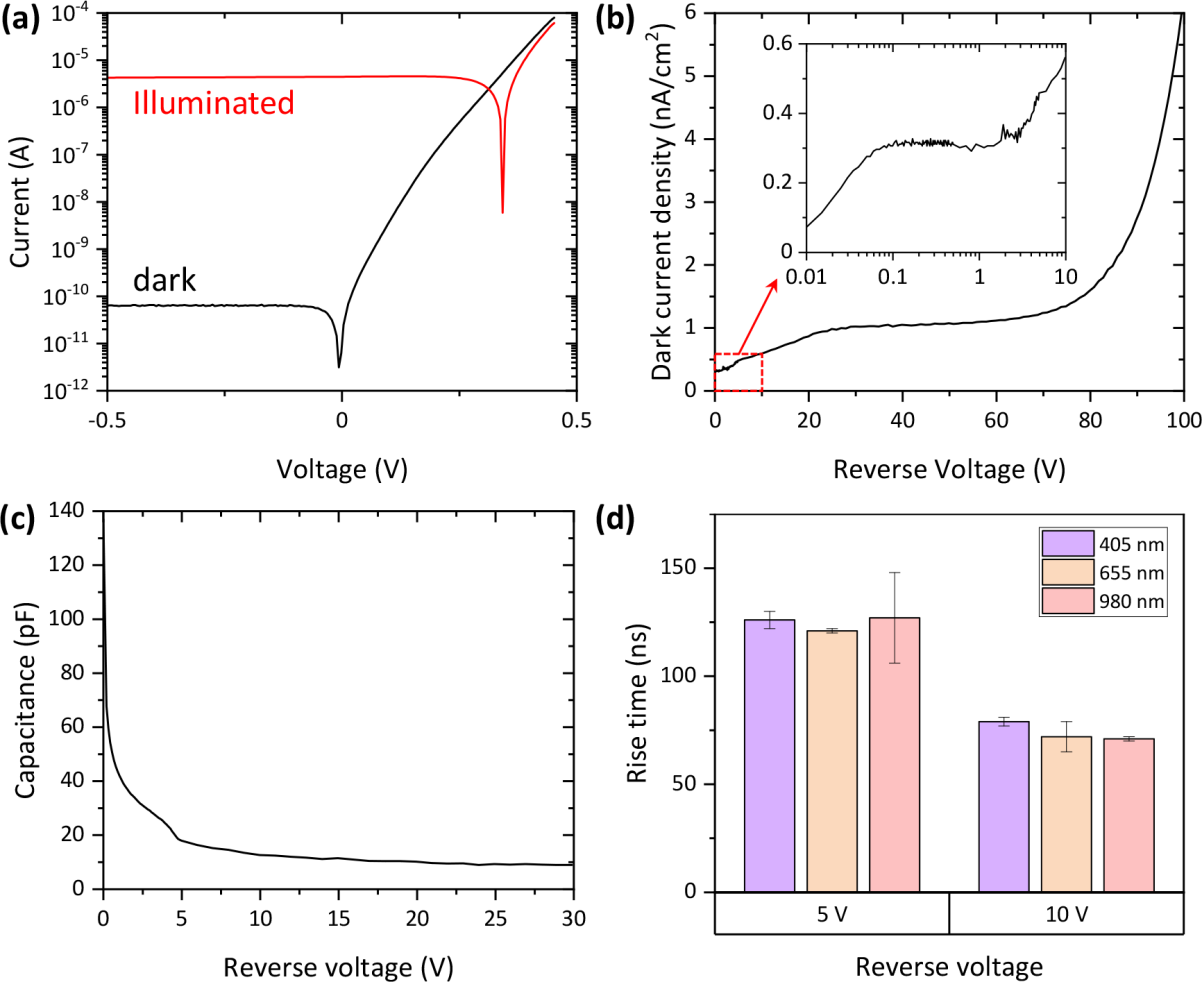
(a) Comparison of IV-characteristics in dark and under continuous room light illumination. (b) Measured dark current density (c) and capacitance of the B-implanted b-Si photodiode as a function of reverse bias voltage. (d) Measured rise times with different biases and illumination wavelengths.

In addition to spectral responsivity, there are multiple other figures of merit that determine the quality of a photodiode. Figure 3a shows the *I*–*V* characteristics of our device in the dark and under room light illumination. A significant difference in current can be seen under these two conditions, indicating a possibility for a highly sensitive operation. Figure 3b further focuses on the dark current density (*J*_d_) with increasing reverse bias voltages. At 10 mV reverse voltage, *J*_d_ is 0.07 nA/cm^2^ and remains below ∼0.3 nA/cm^2^ up to 3 V reverse bias. After that, *J*_d_ steadily increases until 30 V, which is the depletion voltage of the detector. The increase can be explained by the growth of the depletion region, and consequently collection volume, until full depletion is reached. Above 30 V *J*_d_ remains ∼1 nA/cm^2^ until 80 V bias at which the junction breakdown starts to take place. These *J*_d_ values are comparable to the reference photodiodes (e.g., ∼0.1 nA/cm^2^ at −1 V^24,25^) and further demonstrate the good quality of the junction.

Specific detectivity describes the sensitivity of a detector, i.e., its capability to detect small light intensities. To reach the best possible sensitivity, it is necessary to minimize noise in the photodiode. In reverse biased detector, majority of the noise consists of shot noise, which arises from statistical fluctuation of the current in the detector.^20,21^ Considering the dark current as the only noise source, in our devices the specific detectivities at −3 V bias are 1.49 × 10^13^ and 7.30 × 10^13^ Jones for 200 and 1000 nm (λ_peak_, wavelength of peak responsivity), respectively. The values are on the same level as the reference doped pn detectors. For reference, commercial UV photodiode 1 has detectivities of 8.79 × 10^12^ and 4.11 × 10^13^ Jones at 200 nm and λ_peak_ = 960 nm, respectively.^23^ Commercial UV photodiode 2 instead has detectivities of 1.82 × 10^13^ and 7.20 × 10^13^ Jones at 200 nm and λ_peak_ = 970 nm, respectively.^24^ Note that these values were calculated from zero bias Noise Equivalent Power and responsivity curves given in the photodiode datasheets.

Rise time is another important detector parameter, which is defined as the time it takes for the output signal to rise from 10% to 90% of its final value. Rise time determines the speed of the detector and is thus very important factor in certain high-speed applications, such as telecommunication. Capacitance directly impacts the RC time constant and consequently the rise time of the detector.^26^ Capacitance of the B-implanted b-Si photodiodes is 137 pF at zero bias and saturates to 9 pF at the depletion voltage of 30 V (Figure 3c). Rise times of our device are reported in Figure 3d and are ∼120 and ∼75 ns at 5 and 10 V reverse biases, respectively. By increasing the bias, we would expect to further decrease the rise time. The detectors are relatively fast already, but factors that limit the speed will be further discussed later. Finally, the wavelength of the incoming light does not seem to have a major impact on the rise times.

## Discussion

Boron-implanted b-Si detector provides a large improvement in UV responsivity compared to conventionally doped planar Si pn photodiodes. Minimal reflection from the nanostructured surface combined with low-recombination junction and surface allows near ideal performance at UV wavelengths 200–400 nm as well as outside UV all the way up to 1000 nm. Additionally, we expect the performance to improve even further below 200 nm due to carrier multiplication phenomenon. In fact, due to this effect, the spectral responsivity of a truly ideal Si photodiode would exceed one electron per photon at ∼350 nm and below.^27−29^ Thus, there still remains some room for improvement in our photodiode. Nevertheless, the results prove that the excellent EQE previously demonstrated with solar cells^10^ can be achieved with photodiodes, too. The near ideal responsivity over a wide wavelength range also makes the boron-implanted b-Si photodiode a promising candidate for a predictable quantum efficient detector.^30^

The biggest difference between our device and the commercial doped junction photodiodes emerges from the widely dissimilar reflectance. Each reference device has a planar surface, which inevitably results in some reflected light, although having been optimized for UV light detection. Achieving high responsivity between 200 and 400 nm has traditionally been complicated due to highly variable refractive indices of Si, which makes optimizing antireflective coatings difficult.^31^ Furthermore, outside UV, such coatings instead hinder the performance. Hence, eliminating reflectance over a wide wavelength range with AR coatings is extremely challenging.

Another obstacle in UV light detection has been the shallow penetration depth of the UV photons. Charge carriers created near the surface are prone to surface recombination, while the external dopant atoms forming the junction simultaneously increase their probability for Auger recombination. Hence, it has been desirable to move the charge collection region as close to the detector surface as possible. Two methods for that have traditionally been (i) fabrication of ultrashallow junction photodiodes^5,32,33^ and (ii) induction of additional electric field to the detector surface via gradient doping or charged films to drive the charge carriers to the depletion region before recombination.^4,34^ Such methods can solve the recombination problem, but require additional techniques for reduction of reflectance. Another challenge with shallow junctions is their high sheet resistance (several kΩ/sq), which in turn limits the speed of the detector.^4,35^ In our device, the junction is rather deep (∼1.5 μm), resulting in sheet resistance of 85 Ω/sq, which is low enough to not have an impact on the speed. Most importantly, the junction causes no major recombination due to low enough dopant densities throughout it. Recombination is additionally suppressed by generating an additional electric field near the surface through negatively charged Al_2_O_3_ passivation film.

Another recent method for increasing responsivity in Si photodiodes has been forming the junction by inducing an inversion layer on the surface instead of introducing dopant atoms to form the junction.^23,36,37^ This approach also allows utilization of b-Si while minimizing the recombination and has been shown to achieve excellent responsivity from 200 to 1000 nm, e.g., 0.21 A/W at 200 nm. The junction recombination in an induced junction b-Si device has, in fact, been shown to be smaller than in an implanted b-Si junction, similar to one in this work (emitter saturation current of 3 vs 33 fA/cm^2^).^10^ However, an induced junction can lead to higher response times due to high sheet resistance (∼9 kΩ/sq) as well as worse linearity at high input powers.^18,23^ The high sheet resistance creates an additional RC time component, which can create a fundamental limit for an achievable rise time. Compared to a doped pn junction photodiode, the increase would be ∼10 ns at −10 V bias (calculated based on an empirical formula presented in ref (18)) in a device with similar dimensions to ours. In this specific case, the difference is rather small, but in a high-speed photodiode, the high resistivity layer could dominate the rise time. In this work, we have shown that the benefits of the induced junction photodiode, e.g., extremely high responsivity and low junction recombination, can be achieved with traditional doping methods while avoiding the downsides of the induced junction. Furthermore, incorporating B-implanted b-Si photodiodes into established processing lines is simple, as their fabrication largely follows the processing steps of a traditional photodiode. The only additional step is the RIE etching of the b-Si.

In addition to high responsivity, the dark current of our device is very low. Minimal dark current decreases the magnitude of the lowest detectable light intensity, thus making the detector more sensitive. There are multiple factors that explain the low leakage current of our device. First, as mentioned, recombination inside the junction and on the surface of the active area is minimal due to optimized implantation parameters and effective ALD Al_2_O_3_ surface passivation, respectively. Second, Al-nealed SiO_2_ between the anode and the guard ring provides good surface passivation and induces a channel-stopper region between the contacts. Third, the guard ring collects the unwanted currents originating from outside of the active area. Finally, bulk recombination is negligible due to high quality Si substrates used in this work.

In this work, the goal was not to prepare ultrafast detectors. Nevertheless, the rise times are relatively low already and the detector could be made even faster by tuning its design. Our detectors are rather large in area and thus have a high capacitance, which slows down the detector. To minimize the rise time, we could make the detectors smaller and thinner. A smaller active area reduces the capacitance and dark current, and with thinner detectors, it is easier to reach full depletion and eliminate some time components that impact rise time. In this case, the dominating time component would be drift time, which is again tunable by using higher reverse voltages. Such modifications should not impact the responsivity. Nevertheless, based on the calculations made using a theoretical equation for rise time,^26^ we would expect to achieve a below 20 ns rise time with >40 V reverse biases with the current sized (⌀ 5 mm) detectors, too. And as seen in Figure 3b, the leakage currents, although increasing, remain moderate at high biases. Thus, applying a high reverse bias should not impact the sensitivity much either.

## Conclusion

We have fabricated a high-performance Si PIN photodiode utilizing b-Si and an optimized boron-implanted junction, a design originally developed for solar cells. Near-ideal responsivity was achieved from 200 to 1000 nm. More specifically, we obtained responsivities of 0.15–0.30 A/W in UV (200–400 nm), which is a sizable improvement (∼50% on average) to commercial doped junction Si photodiodes. Simultaneously, the dark current of our device remained low (below ∼0.3 nA/cm^2^ up to 3 V reverse bias), allowing sensitive detection. Indeed, the calculated specific detectivities at −3 V bias voltage were 1.49 × 10^13^ and 7.30 × 10^13^ Jones for 200 and 1000 nm, respectively. Finally, unlike in shallow junction photodiodes typically used for UV detection or induced junction photodiodes known for their excellent responsivity over a wide spectral range, the speed of our device is not limited by its sheet resistance. Thus, by tuning the photodiode dimensions and bulk resistivity, an even higher speed should be attainable with this detector design. Our device demonstrates that reaching high responsivities at wavelengths all the way down to UV is possible with conventional photodiode fabrication methods and without trade-offs in other performance characteristics.

## References

[ref1] Monroy E.; Calle F.; Pau J. L.; Muñoz E.; Omnès F.; Beaumont B.; Gibart P. AlGaN-Based UV Photodetectors. J. Cryst. Growth 2001, 230 (3–4), 537–543. 10.1016/S0022-0248(01)01305-7.

[ref2] Chen H.; Liu K.; Hu L.; Al-Ghamdi A. A.; Fang X. New Concept Ultraviolet Photodetectors. Mater. Today (Kidlington) 2015, 18 (9), 493–502. 10.1016/j.mattod.2015.06.001.

[ref3] Zürch M.; Foertsch S.; Matzas M.; Pachmann K.; Kuth R.; Spielmann C. Cancer Cell Classification with Coherent Diffraction Imaging Using an Extreme Ultraviolet Radiation Source. J. Med. Imaging (Bellingham) 2014, 1 (3), 031008 10.1117/1.JMI.1.3.031008. 26158049 PMC4478871

[ref4] Shi L.; Nihtianov S. Comparative Study of Silicon-Based Ultraviolet Photodetectors. IEEE Sens. J. 2012, 12 (7), 2453–2459. 10.1109/JSEN.2012.2192103.

[ref5] Wan X.; Xu Y.; Guo H.; Shehzad K.; Ali A.; Liu Y.; Yang J.; Dai D.; Lin C.-T.; Liu L.; Cheng H.-C.; Wang F.; Wang X.; Lu H.; Hu W.; Pi X.; Dan Y.; Luo J.; Hasan T.; Duan X.; Li X.; Xu J.; Yang D.; Ren T.; Yu B. A Self-Powered High-Performance Graphene/Silicon Ultraviolet Photodetector with Ultra-Shallow Junction: Breaking the Limit of Silicon?. Npj 2D Mater. Appl. 2017, 1 (1), 1–8. 10.1038/s41699-017-0008-4.

[ref6] Repo P.; Benick J.; Vähänissi V.; Schön J.; von Gastrow G.; Steinhauser B.; Schubert M. C.; Hermle M.; Savin H. N-Type Black Silicon Solar Cells. Energy Procedia 2013, 38, 866–871. 10.1016/j.egypro.2013.07.358.

[ref7] Kafle B.; Schön J.; Fleischmann C.; Werner S.; Wolf A.; Clochard L.; Duffy E.; Hofmann M.; Rentsch J. On the Emitter Formation in Nanotextured Silicon Solar Cells to Achieve Improved Electrical Performances. Sol. Energy Mater. Sol. Cells 2016, 152, 94–102. 10.1016/j.solmat.2016.03.031.

[ref8] Pasanen T.; Vähänissi V.; Theut N.; Savin H. Surface Passivation of Black Silicon Phosphorus Emitters with Atomic Layer Deposited SiO2/Al2O3 Stacks. Energy Procedia 2017, 124, 307–312. 10.1016/j.egypro.2017.09.304.

[ref9] Fung T. H.; Pasanen T. P.; Zhang Y.; Soeriyadi A.; Vähänissi V.; Scardera G.; Payne D.; Savin H.; Abbott M. Improved Emitter Performance of RIE Black Silicon through the Application of In-Situ Oxidation during POCl3 Diffusion. Sol. Energy Mater. Sol. Cells 2020, 210, 110480 10.1016/j.solmat.2020.110480.

[ref10] Chen K.; Setälä O. E.; Radfar B.; Kroth U.; Vähänissi V.; Savin H. Harnessing Carrier Multiplication in Silicon Solar Cells Using UV Photons. IEEE Photonics Technol. Lett. 2021, 33 (24), 1415–1418. 10.1109/LPT.2021.3124307.

[ref11] Kim N.; Choi D.; Kim H.; Um H.-D.; Seo K. Silicon Microwire Arrays with Nanoscale Spacing for Radial Junction C-Si Solar Cells with an Efficiency of 20.5. ACS Nano 2021, 15 (9), 14756–14765. 10.1021/acsnano.1c04585. 34583468

[ref12] Repo P.; Haarahiltunen A.; Sainiemi L.; Yli-Koski M.; Talvitie H.; Schubert M. C.; Savin H. Effective Passivation of Black Silicon Surfaces by Atomic Layer Deposition. IEEE j. photovolt. 2013, 3 (1), 90–94. 10.1109/JPHOTOV.2012.2210031.

[ref13] von Gastrow G.; Ortega P.; Alcubilla R.; Husein S.; Nietzold T.; Bertoni M.; Savin H. Recombination Processes in Passivated Boron-Implanted Black Silicon Emitters. J. Appl. Phys. 2017, 121 (18), 185706 10.1063/1.4983297.

[ref14] Cuevas A.; Basore P. A.; Giroult-Matlakowski G.; Dubois C. Surface Recombination Velocity of Highly Doped n-type Silicon. J. Appl. Phys. 1996, 80 (6), 3370–3375. 10.1063/1.363250.

[ref15] Kerr M. J.; Cuevas A. Very low bulk and surface recombination in oxidized silicon wafers. Semicond. Sci. Technol. 2002, 17 (1), 35 10.1088/0268-1242/17/1/306.

[ref16] Setälä O. E.; Pasanen T. P.; Ott J.; Vähänissi V.; Savin H. Al-neal Degrades Al_2_O_3_ Passivation of Silicon Surface. Phys. Status Solidi (a) 2021, 218 (23), 2100214 10.1002/pssa.202100214.

[ref17] Chen K.; Setälä O. E.; Liu X.; Radfar B.; Pasanen T. P.; Serué M. D.; Heinonen J.; Savin H.; Vähänissi V. Excellent Responsivity and Low Dark Current Obtained with Metal-Assisted Chemical Etched Si Photodiode. IEEE Sens. J. 2023, 23 (7), 6750–6756. 10.1109/JSEN.2023.3246505.

[ref18] Heinonen J.; Haarahiltunen A.; Vähänissi V.; Pasanen T. P.; Savin H. I.; Juntunen M. A. Effect of Anode Sheet Resistance on Rise Time of Black Silicon Induced Junction Photodiodes. Proc. SPIE 11997, Optical Components and Materials XIX 2022, San Francisco, CA, U.S.A., 4 March 2022, SPIE, 2022; Vol. 11997, pp 74–82. 10.1117/12.2609632.

[ref19] Ayedh H. M.; Forbom C. W.; Heinonen J.; Rauha I. T. S.; Yli-Koski M.; Vähänissi V.; Savin H. Fast Wafer-Level Characterization of Silicon Photodetectors by Photoluminescence Imaging. IEEE Trans. Electron Devices 2022, 69 (5), 2449–2456. 10.1109/TED.2022.3159497.

[ref20] Gong X.; Tong M.; Xia Y.; Cai W.; Moon J. S.; Cao Y.; Yu G.; Shieh C.-L.; Nilsson B.; Heeger A. J. High-Detectivity Polymer Photodetectors with Spectral Response from 300 Nm to 1450 Nm. Science 2009, 325 (5948), 1665–1667. 10.1126/science.1176706. 19679770

[ref21] Liu S.; Wei Z.; Cao Y.; Gan L.; Wang Z.; Xu W.; Guo X.; Zhu D. Ultrasensitive Water-Processed Monolayer Photodetectors. Chemical Science 2011, 2 (4), 796–802. 10.1039/c0sc00488j.

[ref22] Hoex B.; Gielis J. J. H.; van de Sanden M. C. M.; Kessels W. M. M. On the C-Si Surface Passivation Mechanism by the Negative-Charge-Dielectric Al2O3. J. Appl. Phys. 2008, 104 (11), 113703 10.1063/1.3021091.

[ref23] OSI Optoelectronics. Planar Diffused UV Enhanced Series Datasheet. OSI Optoelectronics, 2022. https://osioptoelectronics.com/Libraries/Datasheets/UV-Planar-Diffused-Photodiodes.sflb.ashx (accessed 2022–12–13).

[ref24] Hamamatsu Photonics. Si photodiodes – S1337 Series. Hamamatsu, 2015. https://www.hamamatsu.com/content/dam/hamamatsu-photonics/sites/documents/99_SALES_LIBRARY/ssd/s1337_series_kspd1032e.pdf (accessed 2022–12–13).

[ref25] Opto Diode. Photodiodes: UV Enhanced Detectors (UVG) - UVG100. Opto Diode, 2019. https://optodiode.com/pdf/UVG100DS.pdf (accessed 2022–12–13).

[ref26] Goushcha A. O.; Tabbert B. On response time of semiconductor photodiodes. Opt. Eng. 2017, 56 (9), 097101 10.1117/1.OE.56.9.097101.

[ref27] Scholze F.; Rabus H.; Ulm G. Mean Energy Required to Produce an Electron-Hole Pair in Silicon for Photons of Energies between 50 and 1500 EV. J. Appl. Phys. 1998, 84 (5), 2926–2939. 10.1063/1.368398.

[ref28] Scholze F.; Henneken H.; Kuschnerus P.; Rabus H.; Richter M.; Ulm G. Determination of the Electron–Hole Pair Creation Energy for Semiconductors from the Spectral Responsivity of Photodiodes. Nucl. Instrum. Methods Phys. Res. A 2000, 439 (2–3), 208–215. 10.1016/S0168-9002(99)00937-7.

[ref29] Shi L.; Nihtianov S.; Scholze F.; Gottwald A.; Nanver L. K. High-Sensitivity High-Stability Silicon Photodiodes for DUV, VUV and EUV Spectral Ranges. In Proc. SPIE 8145, UV, X-Ray, and Gamma-Ray Space Instrumentation for Astronomy XVII 2011, San Diego, California, United States, 13 September 2011, SPIE, 2011; Vol. 8145, pp 219–227. 10.1117/12.891865.

[ref30] Sildoja M.; Manoocheri F.; Merimaa M.; Ikonen E.; Müller I.; Werner L.; Gran J.; Kübarsepp T.; Smîd M.; Rastello M. L. Predictable Quantum Efficient Detector: I. Photodiodes and Predicted Responsivity. Metrologia 2013, 50 (4), 385 10.1088/0026-1394/50/4/385.

[ref31] Hamden E. T.; Greer F.; Hoenk M. E.; Blacksberg J.; Dickie M. R.; Nikzad S.; Martin D. C.; Schiminovich D. Ultraviolet antireflection coatings for use in silicon detector design. Appl. Opt. 2011, 50 (21), 4180–4188. 10.1364/AO.50.004180. 21772406

[ref32] Nikzad S.; Jones T. J.; Elliott S. T.; Cunningham T. J.; Deelman P. W.; Walker A. B. C. II; Oluseyi H. M. Ultrastable and Uniform EUV and UV Detectors. In Proc. SPIE 4139, Instrumentation for UV/EUV Astronomy and Solar Missions 2000, San Diego, CA, United States, 18 December 2000, SPIE; 2000, Vol. 4139, pp 250–258. 10.1117/12.410541.

[ref33] Shi L.; Sarubbi F.; Nanver L. K.; Kroth U.; Gottwald A.; Nihtianov S. Optical Performance of B-Layer Ultra-Shallow-Junction Silicon Photodiodes in the VUV Spectral Range. Procedia Eng. 2010, 5, 633–636. 10.1016/j.proeng.2010.09.189.

[ref34] Xia Z.; Zang K.; Liu D.; Zhou M.; Kim T.-J.; Zhang H.; Xue M.; Park J.; Morea M.; Ryu J. H.; Chang T.-H.; Kim J.; Gong S.; Kamins T. I.; Yu Z.; Wang Z.; Harris J. S.; Ma Z. High-Sensitivity Silicon Ultraviolet p+-i-n Avalanche Photodiode Using Ultra-Shallow Boron Gradient Doping. Appl. Phys. Lett. 2017, 111 (8), 081109 10.1063/1.4985591.

[ref35] Xia S.; Sarubi F.; Naulaerts R.; Nihtianov S.; Nanver L. Response Time of Silicon Photodiodes for DUV/EUV Radiation. In IEEE Instrumentation and Measurement Technology Conference 2008, Victoria, BC, Canada, 12–15 May 2008, IEEE, 2008; pp 1956–1959. 10.1109/IMTC.2008.4547368.

[ref36] Juntunen M. A.; Heinonen J.; Vähänissi V.; Repo P.; Valluru D.; Savin H. Near-Unity Quantum Efficiency of Broadband Black Silicon Photodiodes with an Induced Junction. Nat. Photonics 2016, 10 (12), 777–781. 10.1038/nphoton.2016.226.

[ref37] Garin M.; Heinonen J.; Werner L.; Pasanen T. P.; Vähänissi V.; Haarahiltunen A.; Juntunen M. A.; Savin H. Black-Silicon Ultraviolet Photodiodes Achieve External Quantum Efficiency above 130. Phys. Rev. Lett. 2020, 125 (11), 117702 10.1103/PhysRevLett.125.117702. 32976002

